# Ecology of cold environments: new insights of bacterial metabolic adaptation through an integrated genomic-phenomic approach

**DOI:** 10.1038/s41598-017-00876-4

**Published:** 2017-04-12

**Authors:** Stefano Mocali, Carolina Chiellini, Arturo Fabiani, Silvia Decuzzi, Donatella de Pascale, Ermenegilda Parrilli, Maria Luisa Tutino, Elena Perrin, Emanuele Bosi, Marco Fondi, Angelina Lo Giudice, Renato Fani

**Affiliations:** 1 Consiglio per la ricerca in agricoltura e l’analisi dell’economia agraria – Centro di Ricerca per l’Agrobiologia e la Pedologia (CREA-ABP), via di Lanciola 12/A, 50125 Firenze, Italy; 2 Institute of Protein Biochemistry, CNR, Via Pietro Castellino 111, 80131 Naples, Italy; 3 Department of Chemical Sciences, University of Naples ‘Federico II’, Complesso Universitario, Monte Sant’Angelo, Via Cinthia 4, 80126 Naples, Italy; 4 Department of Biology, LEMM, Laboratory of Microbial and Molecular Evolution Florence, University of Florence, I-50019 Sesto Fiorentino (FI), Italy; 5 Institute for the Coastal Marine Environment, National Research Council (IAMC-CNR), Spianata San Raineri 86, 98122 Messina, Italy; 6 Department of Chemical, Biological, Pharmaceutical and Environmental Sciences, University of Messina, Viale F. Stagno d’Alcontrès 31, 98166 Messina, Italy

## Abstract

Cold environments dominate Earth’s biosphere, hosting complex microbial communities with the ability to thrive at low temperatures. However, the underlying molecular mechanisms and the metabolic pathways involved in bacterial cold-adaptation mechanisms are still not fully understood. Herein, we assessed the metabolic features of the Antarctic bacterium *Pseudoalteromonas haloplanktis* TAC125 (PhTAC125), a model organism for cold-adaptation, at both 4 °C and 15 °C, by integrating genomic and phenomic (high-throughput phenotyping) data and comparing the obtained results to the taxonomically related Antarctic bacterium *Pseudoalteromonas* sp. TB41 (PspTB41). Although the genome size of PspTB41 is considerably larger than PhTAC125, the higher number of genes did not reflect any higher metabolic versatility at 4 °C as compared to PhTAC125. Remarkably, protein S-thiolation regulated by glutathione and glutathionylspermidine appeared to be a new possible mechanism for cold adaptation in PhTAC125. More in general, this study represents an example of how ‘multi-omic’ information might potentially contribute in filling the gap between genotypic and phenotypic features related to cold-adaptation mechanisms in bacteria.

## Introduction

About one-half of global primary production occurs in the open ocean^1, 2^, where most of the organic matter turnover is regulated by microorganisms^3^. As a result, a large fraction of the carbon flux patterns, which affect ecosystem functioning and climate change, responds to microbial metabolism. As oceans are permanently colder than 5 °C below a depth of 1000 m^4^, diverse cold-adapted (*psychrophilic*) microorganisms have evolved a number of adaptive strategies in order to maintain their vital metabolic functions under such permanent severe conditions^5, 6^. However, temperature is not the only factor that controls the typical traits of psychrophilic bacteria and it has been argued that the use of temperature-dependent growth rate as a metric for cold-adaptation could provide severe misconceptions^7^. In fact, the ability of an organism to survive and grow under cold conditions is dependent on a number of adaptive strategies evolved in order to successfully counteract additional stress factors associated with cold environments, such as radiations, excessive UV, high or low pH, high osmotic pressure and low nutrient availability^6, 8, 9^. Thus, it is actually difficult to infer general principles that may explain the capacity for many psychrophiles to adapt their genomic and metabolic features to their native cold habitats.

Most of the available data on psychrophilic adaptation results from previous studies of proteins and genes, and from physiological analysis of individual strains^4, 6, 10^. In recent years, the availability of a large number of genomes of psychrophilic organisms and mesophilic phylogenetic relatives has strongly stimulated comparative genomic analyses, which have provided significant insights into the ecological fitness traits and metabolic versatilities of psychrophiles across different temperature ranges^11–13^ or ecological niches^14^. Furthermore, other “omic” technologies, such as transcriptomics, proteomics, metabolomics and metagenomics have been used to study the differential expression of genes, proteins or functions in microorganisms grown under different cold temperatures^9, 11, 12, 15–18^. The overall results suggested that the psychrophilic lifestyle is most likely conferred not by a unique set of genes but by a collection of synergistic changes in both genome content and metabolic features^9, 19, 20^. Moreover, inter-species and inter-strain differences must be also taken into account when developing a global picture of the mechanisms employed for cold adaptation. Thus, it is clear that different strategies are used by different organisms to survive at low temperatures.

In this context, the Antarctic bacterium *Pseudoalteromonas haloplanktis* TAC125 (PhTAC125) is one of the most intensively investigated psychrophiles^21^, due to its extremely fast growth and its wide temperature range (from −2.5 to 25 °C). Thus, it’s not surprising that this organism was the first Antarctic marine bacterium whose genome was fully sequenced and annotated^20^. This, combined with its remarkable versatility and its biotechnological potential^22–25^ makes PhTAC125 an interesting and potentially useful model for investigating strategies adopted by psychrophilic bacteria to survive at low temperatures. Furthermore, its proteome^26, 27^, the structural features of its membrane^28, 29^, detailed growth phenotypes^30, 31^ and a genome-scale metabolic reconstruction^32^ determined under different temperatures are also available. Nevertheless, many aspects of its adaptation to cold are still unclear. In fact, little is known about the phenotypic features of PhTAC125 expressed at low temperatures (and at the whole cellular level) despite the importance of linking genome functionality and source environmental niche. This is even more important if we consider that a large fraction of the genes from genomic sequencing analysis have no ascribed function and even genes with ascribed functions are primarily based on DNA sequence homology, with little or no direct support of experimental data^33^.

In this work we exploited the metabolic profile of the “model organism” PhTAC125 grown at 4 °C and 15 °C by means of a combined genomic-phenomic approach, and compared the data to the closely-related *Pseudoalteromonas* sp. TB41 (PspTB41), an Antarctic bacterium highly efficient in the production of numerous secondary metabolites, some of which active against pathogens^34^ and recently proposed to be affiliated to the *P*. *haloplanktis* species on the basis of genome-scale phylogenetic and DNA composition analysis^35^. We have chosen these two strains because, in spite of the fact that they are phylogenetically related, they have been isolated from different ecological niches in Antarctica (seawater and a marine sponge, respectively) and display a remarkable variability in their genome size (3.85 and 4.63 Mb for PhTAC125 and PspTB41, respectively). This latter point might, in principle, suggest the existence of different metabolic features related to cold adaptation. To the best of our knowledge, no work concerning a massive exploitation of the overall phenotypic features (phenome) of the metabolism of cold-adapted bacteria (or other extremophiles) grown at different temperatures has been carried out so far.

## Results

### General genomic features and comparisons

Considering the genome size, there are notable differences between PhTAC125 and PspTB41 (3850272 bp and 4632606 bp, respectively), with the latter genome that is about 800,000 bp larger than that of PhTAC125. Accordingly, the PspTB41 genome possesses more genes than PhTAC125 (4127 *vs* 3484), which might suggest a higher genetic variety and, in turn, a higher metabolic versatility. The comparative analysis of the two genomes revealed that the two strains share 2727 genes and that PspTB41 has almost the double of unique genes than PhTAC125 (1490 *vs* 757), consistently with the different genome size of these bacteria.

**Figure 1:**
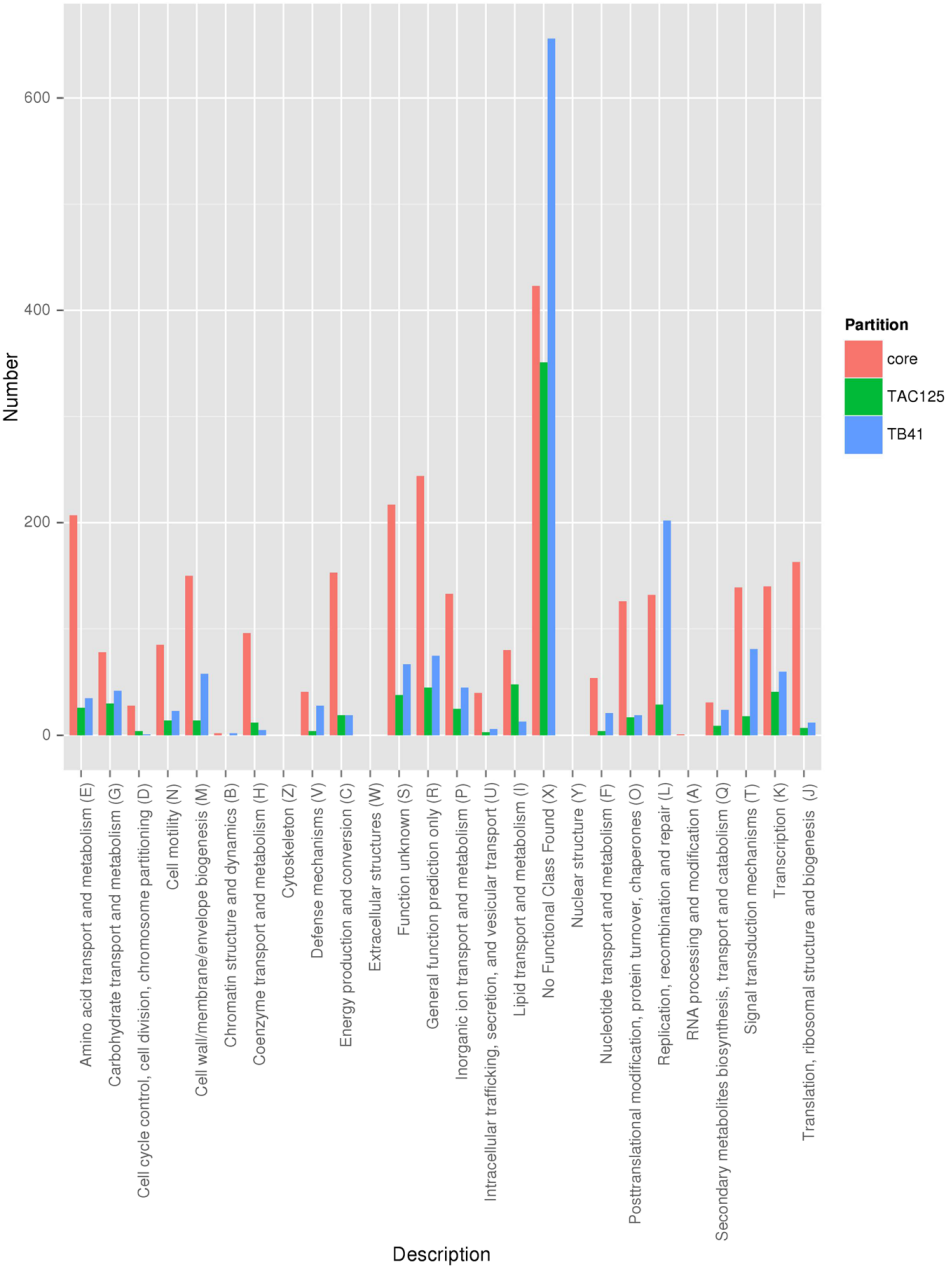
Frequency (%) of the core and specific genes associated to COG categories in the Antarctic bacteria PhTAC125 and PspTB41 based on genomic data. COG functional categories: RNA processing and modification (A); Chromatin structure and dynamics (B); Energy production and conversion (C); Cell cycle control, cell division, chromosome partitioning (D); Amino acid transport and metabolism (E); Nucleotide transport and metabolism (F); Carbohydrate transport and metabolism (G); Coenzyme transport and metabolism (H); Lipid transport and metabolism (I); Translation, ribosomal structure and biogenesis (J); Transcription (K); Replication, recombination and repair (L); Cell wall/membrane/envelope biogenesis (M); Cell motility (N); Posttranslational modification, protein turnover, chaperones (O); Inorganic ion transport and metabolism (P); Secondary metabolites biosynthesis, transport and catabolism (Q); General function prediction only (R); Function unknown (S); Signal transduction mechanisms (T); Intracellular trafficking, secretion, and vesicular transport (U); Defense mechanisms (V); Extracellular structures (W); No Functional Class Found (X); Nuclear structure (Y); Cytoskeleton (Z).

Even though the COG functional analysis performed on the genome of the two strains revealed many differences in COG distributions (Fig. 1), nevertheless both genomes exhibited almost a 60% of genes annotated with unknown or predicted functions (COG X category). PspTB41 displayed a high amount of genes belonging to the COG L group (‘Replication, recombination and repair’). Since the L category embeds also Mobile Genetic Elements (MGEs), we checked the presence of such elements in the genome of the two bacteria. Data obtained (not shown) revealed that PspTB41 genome embeds almost 200 MGEs more than PhTAC125. iii) Another interesting and striking difference between the two genomes concerned the I category (lipid transport and metabolism), which was enriched in the PhTAC125 unique genome.

### General Phenomic Features

**Figure 2:**
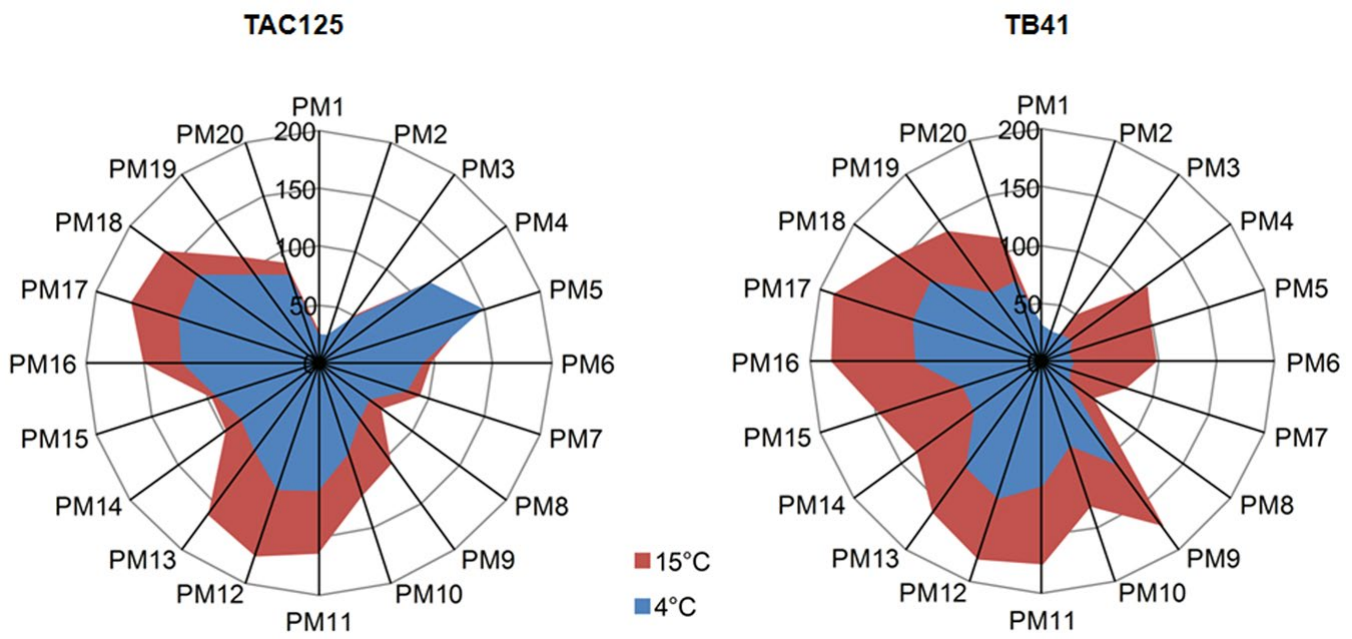
Phenotype Microarray (PM) experiment. Radar plots representing the number of the different growth phenotypes of the PhTAC125 and PspTB41 strains as assessed by the PM experiment at 4 °C (blue) and 15 °C (red). Each radial strip corresponds to a single PM microplate (PM1-20). PM categories: Carbon Sources (PM1, PM2), Nitrogen Sources (PM3), Phosphorus and Sulfur Sources (PM4), Nutrient Supplements (PM5), Peptide Nitrogen sources (PM6, PM7, PM8), Osmolytes (PM9), pH (PM10) and Chemicals (PM11-20).

All the Phenotype Microarray (PM) raw data are available as additional information to this paper (Table S2). By comparing the binary results of the PM reactions (growth/no-growth) of PhTAC125 and PspTB41 expressed across the complete PM array under 4 and 15 °C, interesting differences were observed. The results showed that both strains displayed a remarkable difference of their metabolic potential (the range of degraded compounds) at 4 °C and 15 °C, exhibiting a strong reduction of cellular metabolism at the lowest temperature (Fig. 2). Nevertheless, it should be noted that the decrease of metabolic activity from 15 °C to 4 °C was higher in PspTB41 than PhTAC125 (42.5% and 8.7%, respectively). Indeed, PhTAC125 showed a better response in terms of metabolic activity at 4 °C, especially for conditions comprised onto PM 4–8 plates (Fig. 2), which are related to P-source and S-source metabolisms (PM 4), nutrient supplements (PM 5), peptides and nitrogen sources (PM 6–8).

The PM analysis revealed that the 52% of the PM substrates were likewise used by PhTAC125 at both temperatures whereas no metabolic activity was detected in 37% of the substrates. Thus, the catabolism of 89% of the tested substrates is not affected by temperature, whereas the remaining 11% of substrates were specifically utilized only at either 15 °C or 4 °C (8% and 3%, respectively). In contrast, only the 69% of the metabolic potential of PspTB41 was stable at both temperatures whereas the remaining 31% was differentially utilized at the two temperatures. Thus, the metabolic downshift occurred from 15 to 4 °C revealed by the PM analysis of PhTAC125 was 8.7% (the number of positive phenotypes decreased from 1155 to 1054), whereas in PspTB41 was 42.4% (the number of positive phenotypes decreased from 1194 to 687). Remarkably, additional 27% and 1% of PM substrates were specifically utilized by PspTB41 only at 15 °C and 4 °C, respectively, indicating a better metabolic adaptation of PspTB41 strain at 15 °C rather than at 4 °C.

At cold temperature (4 °C) PhTAC125 and PspTB41 revealed a metabolic overlapping in about 71% of the PM substrates, whereas additional 5% and 24% of PM substrates were specifically metabolized only by PspTB41 or PhTAC125, respectively. It should be noted that most of the substrates metabolized by PhTAC125 only at 4 °C belong to the following KEGG categories: “nitrogen peptides” (163 of 465), “nutrient stimulation” (96 of 465), “phosphate and sulfur” (81 of 465), and “chemicals” (71 of 465). Many of the “chemicals” are biocide or antimicrobial compounds, indicating an increased resistance of PhTAC125 to such molecules at low temperatures. A list with the “unique” substrates specifically used by PhTAC125 and PspTB41 only at 4 °C is reported as additional information (Tables S3 and S4, respectively).

**Figure 3:**
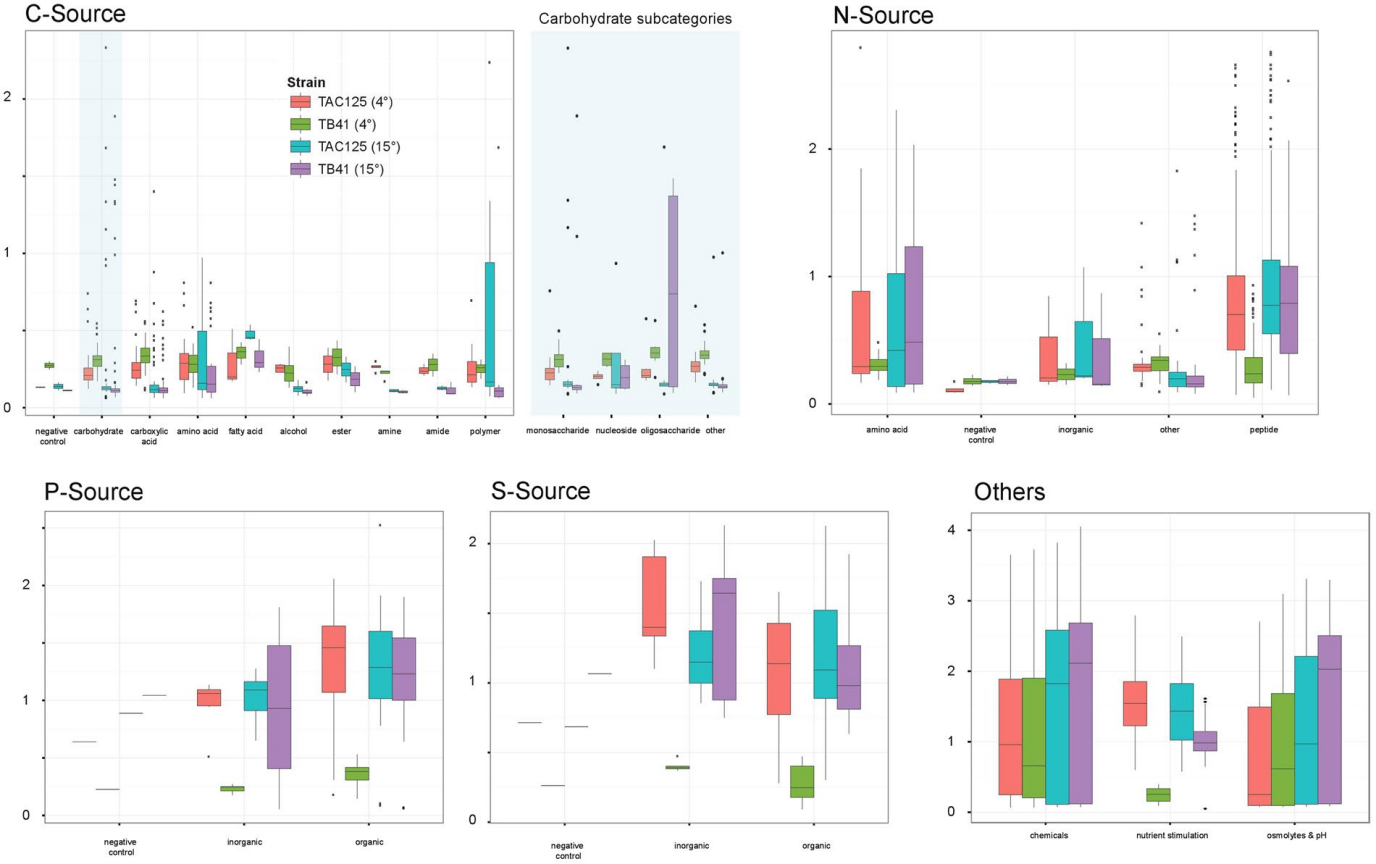
The utilization of carbon (C), nitrogen (N) phosphorous (P) and sulfur (S) sources under 4 °C and 15 °C. A boxplot displaying the PM 4–8 source utilization. The figures include all positive phenotypes (gray circles) for PhTAC125 and PspTB41 (at 4 °C and 15 °C). The carbohydrate group was further divided into sub-types.

In order to more directly compare the ability of the two strains to utilize specific sources at 4 and 15 °C, a boxplot for each PM category is reported in Fig. 3. Here, all the phenotypes were grouped based on the type of PM source molecules. Furthermore, the tested carbohydrate source phenotypes were further sub-grouped based on the type of carbon source molecules. Remarkably, at 15 °C PspTB41 exhibited the metabolic ability to use oligosaccharides significantly above the levels observed at 4 °C and compared to PhTAC125 at both temperatures, indicating a less adaptation of the C metabolism of PspTB41 to cold temperature (4 °C), compared to PhTAC125.

The two strains showed a similar metabolic activity on nitrogen compounds at 15 °C. On the other hand, at low temperature (4 °C) PhTAC125 displayed a better response for N-source growth enhancement compared to PspTB41, and it could better utilize many dipeptides and amino acids such as Glutamic acid, Glutamine, Citrulline, Asparagine, Serine, Aspartic acid, Threonine, Alanine and Arginine. Furthermore, also in the case of P and S metabolisms the two strains exhibited similar metabolic profiles at 15 °C but, once again, when grown at 4 °C PspTB41 significantly decreased its metabolic potential compared to PhTAC125. Finally, as PhTAC125 is able to transport long-chain fatty acids across the cell membrane, the fact that PhTAC125 was able to more efficiently utilize fatty-acid molecules (i.e. TWEEN 80) as carbon source than PspTB41 strain is not that surprising.

Overall, whereas PhTAC125 and PspTB41 displayed a similar trend of growth/no-growth response on most of PM substrates at 15 °C, significant differences raised when their metabolic profiles were compared at 4 °C. In particular, under cold temperature PhTAC125 can more effectively utilize nitrogen, sulfur and phosphorous sources than PspTB41, thus suggesting an enhanced ability to synthesize proteins under cold temperatures.

### Phenomic features for cold-adaptation

**Figure 4:**
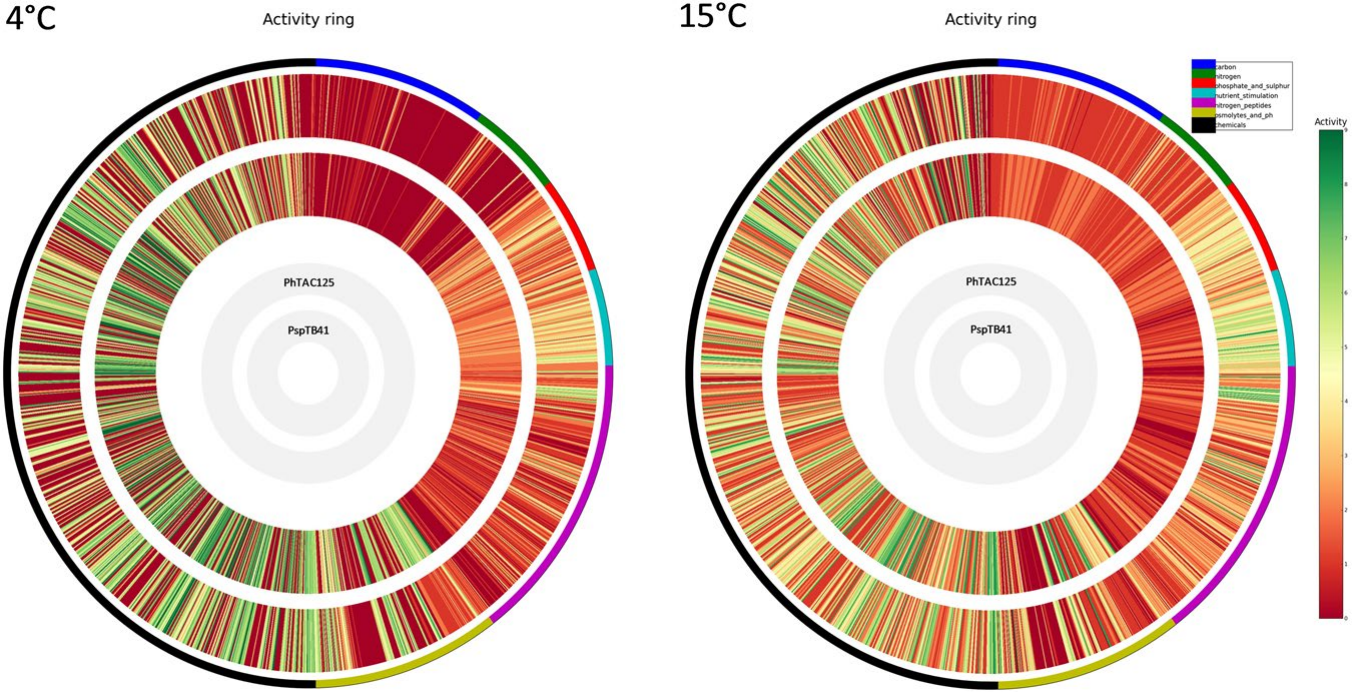
High-throughput metabolic activity of PhTAC125 and PspTB41 at 4 °C and 15 °C. The metabolic activity is expressed as AV value and colored from red (low activity) to green (high activity) and visualized through the DuctApe Activity rings, indicating the PM substrates differentially utilized by PhTAC125 and PspsTB41 at 4 °C and 15 °C. Circles display (from the outside): (1) the metabolic activity of PhTAC125 strain; (2) the metabolic activity of TB41 strain; the lane external to the circles is color-coded according to different functional PM categories: blue indicates Carbon sources (PM1,2); green indicates Nitrogen sources (PM3); red indicates Phosphate and Sulfure substrates (PM4); light blue indicates Nutrient Stimulation substrates (PM5); purple indicates Nitrogen Peptides (PM6,7,8); light green indicates Osmolytes and pH (PM9,10); black indicates Chemical compounds (PM11-20).

Individual differences among each of the 1920 PM metabolic conditions of the two strains were then assessed and represented in detail as circular plots (Activity Rings) (Fig. 4). In this figure, each concentric circle represents one of the two strains whereas each radial strip corresponds to a single tested phenotype. This analysis confirmed the overall phenotypic ability of PhTAC125 to more efficiently grow at 4 °C (Fig. 4a) in presence of several chemical compounds (PM11-20) compared to PspTB41. In fact, PhTAC125 is able to grow at 4 °C onto 75 substrates that PspTB41 cannot use. Conversely, PspTB41 is able to grow only onto 51 substrates that PhTAC125 cannot use. In contrast, when such metabolic potentials are compared at 15 °C, things are different. In fact, at such temperature PhTAC125 is able to grow onto just 26 substrates that PspTB41 cannot use, whereas PspTB41 is able to grow only onto 98 substrates that PhTAC125 cannot use. Thus, PhTAC125 showed an overall higher metabolic potential than PspTB41 to adapt to environmental conditions and chemical stress at 4 °C but not at 15 °C.

To determine what compounds were potentially involved in cold adaptation, we firstly looked for the metabolic response of those substrates with oxidizing properties, which were differentially used at the two temperatures. Interestingly, the resistance to the oxidant Plumbagin is given by *ygfZ* gene, which is involved in chromosome replication initiation and releasing intracellular oxidative stress in *Escherichia coli* ^36^. Remarkably, it was reported that *yfgZ* null mutant of *E*. *coli* grows slowly, especially at low temperature, and it is hypersensitive to oxidative stress^37^. Thus, although *yfgZ* is not an essential gene, these results indicate that it is important for *E*. *coli* proliferation at low temperatures. Moreover, PhTAC125 was able to grow on folate-antagonist compounds (es. Sulfisoxazole, Trimethoprim), suggesting the presence of a *dfrA1* trimethoprim-resistance cassette on its chromosome, as previously reported by Boucher *et al*.^38^, which is essential for *yfgZ* functioning. Such indication was confirmed by scanning the PhTAC125 genome that revealed the presence of both *ygfZ* (PSHAa0722) and *dfrA1* genes (PSHAa2653), which are absent in PspTB41 genome.

An important substrate known to protect PhTAC125 against oxidative stresses generated by low temperatures is Glutathione (GSH), the best known low molecular weight (LMW) thiol and a key component of the cytoplasmic redox buffering capacity^39–41^, which PhTAC125 better utilize as both S-source and nutrient supplement than PspTB41. Furthermore, at 4 °C PhTAC125 is able to grow on 1-Chloro-2,4-Dinitrobenzene (CDNB) that is known to be bound by glutathione S-transferases (GSTs), versatile enzymes playing an important role as detoxifier of exogenous and xenobiotic compounds in many bacteria^42, 43^, including the Antarctic bacteria *Pseudoalteromonas* sp. ANT506, where GST was shown to be a typical cold active enzyme^44^. Remarkably, two GST-like genes (YibF) are present in the PhTAC125 genome but not in PspTB41.

Another key metabolic feature for the cold-adaptation of bacteria is the ability to face the osmotic stress. Thus, we evaluated the ability of the two strains to grow on PM9 plate, which yields information on the bacterial tolerance to osmotic stress. We observed the ability of PhTAC125 to grow at 15 °C under a wide range of NaCl concentrations (1–10%), according to previous results conducted at such temperature^20^, and KCl concentrations (3–6%). Similar results were observed for PspTB41 at 15 °C as well. In remarkable contrast, at 4 °C PhTAC125 is not able to grow under high osmotic stress (NaCl >5%), even if in the presence of a number of Glycine betaines (N,N,N-trimethyl analogs, i.e. glycine, carnitine, trehalose, trigonelline, choline, etc.). Similarly, despite the ability to grow at 4 °C under a wide range of NaCl concentrations (1–8%), PspTB41 exhibited strong grow limitations under potassium chloride (KCl) >4% compared to 15 °C.

### Linking genomic and phenomic data

**Figure 5:**
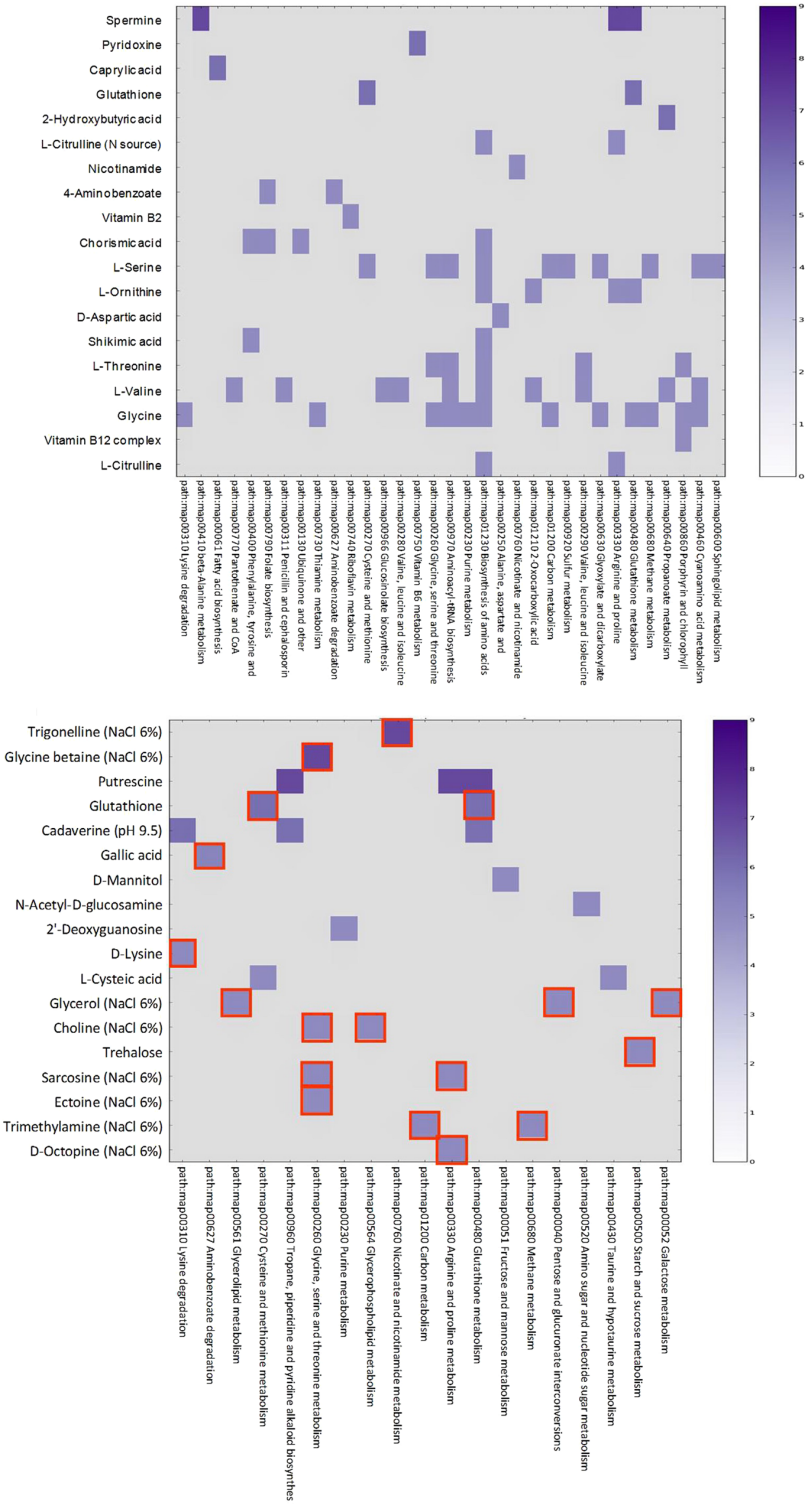
Combined genome/phenome variability of PhTAC125 and PspTB41 (red-border squares) strains at 4 °C (a) and 15 °C (b). The main PM substrates (AV >5) which were differently used by the two strains are reported on the Y axis whereas the metabolic pathways involved are reported on X axis. The color indicates the magnitude of such AV difference (0–9).

Lastly, we identified the number of phenotypic conditions provided by PM plates which resulted ‘linked’ to the genomic variability between PhTAC125 and PspTB41 by means of the DuctApe tool. We performed this analysis with AV values obtained from the PM experiments conducted at both 4 °C and 15 °C in order to highlight which metabolic differences were related to temperature and the results are summarized in Fig. 5a and b, respectively (cut-off: AV ≥5).

At 4 °C, 19 PM substrates differently used by the PhTAC125 and PspTB41 were found to be linked to genomic discrepancies between the two strains, involving a total of 33 different metabolic pathways (Fig. 5a). Most of them belong to the PM category “nutrient stimulators” and are mainly related to Glutathione metabolism (KEGG map480), Arginine and Proline metabolism (KEGG map480), Fatty acid biosynthesis (KEGG map61) and Biosynthesis of amino acids metabolism (KEGG map1230), but also to vitamin B6 metabolism (KEGG map750), which appeared to be affected by cold temperature. In particular, the highest number of genome-related substrates differently used by PhTAC125 and PspTB41 occurred in the ‘Biosynthesis of amino acids’ metabolic pathway (KEGG map1230), thus indicating the amino acid metabolism as one of the key metabolisms involved in cold adaptation response. Spermine (KEGG code C00750) resulted as the substrate with the highest AV value (AV = 7). Another strain-specific substrate apparently related to cold adaptation of PhTAC125 is Pyridoxine (KEGG code C00314), AV = 6 (Fig. 5a), which is involved in vitamin B6 metabolism and was better metabolized by PhTAC125 than PspTB41. Furthermore, the ability of PhTAC125 to grow at 4 °C on Caprylic Acid (KEGG code C06423) (AV = 6) - which is related to fatty acid biosynthesis - is consistent with the predicted higher fluxes of reactions involved in fatty acid biosynthesis/metabolism following a cold shock as reported in a recent paper^32^. Further substrates with relatively high AV value are Glutathione (KEGG code C00051), Hydroxy-Butyric Acid (KEGG code C05984), Citrulline (KEGG code C00327), Nicotinamide (KEGG code C00153) (AV = 6) and Aspartate (KEGG code C00402) (AV = 5), which were more efficiently used by PhTAC125 than by PspTB41. Finally, PhTAC125 better utilized L-Serine (KEGG code C00065), L, Ornithine (KEGG code C00077), L-Threonine (KEGG code C00188), L-Valine (KEGG code C00183) and Glycine (KEGG code C00083) than PspTB41 as N-source or nutritional supplements (Table S2).

On the other hand, relevant metabolic differences were highlighted between PhTAC125 and PspTB41 when the strains were compared at 15 °C (Fig. 5b). For example, Trigonelline (KEGG code C01004) and betaine (KEGG code C00719) showed the highest AV values (AV = 7) in PspTB41 under osmotically stressed conditions (NaCl 6%) which are limiting for PhTAC125; in contrast, Putrescine exhibited the highest AV values (AV = 7) in PhTAC125 within the ‘Arginine and proline metabolism’ (KEGG map330), ‘Glutathione metabolism’ (KEGG map480) and the ‘Tropane, piperidine and pyridine alkaloid biosynthesis’ pathway (KEGG map960).

Furthermore, PspTB41 was able to better catabolize Glutathione and Cadaverine (AV = 6), Gallic Acid (AV = 5.25), D-Mannitol, N-Acetyl-D-Glucosamine, 2′-Deoxy-Guanosine, Hydroxy Glutamic Acid, Hydroxy Glutamate, Glycerol, Choline, D-Trehalose (AV = 5), including a number of substrates added to NaCl 6% such as Glycerol, Choline, Sarcosine, Ectoine, Trimethylamine and Octopine (AV = 5).

Most of the other compounds with AV <5 are similarly catabolized at 4 °C, even if at a lower rate. Thus, they will be not considered involved in the cold-adaptation strategy but as simply related to specific features which differences PhTAC125 and PspTB41.

## Discussion

Linking genome and phenotypic features is one of the most important and challenging tasks in microbial ecology. Here, we have carried out a genome-scale comparison of two taxonomically related Antarctic *P*. *haloplanktis* strains, and linked the data to their high-throughput phenotypic characterization conducted under 4 °C and 15 °C by means of the Phenotype Microarray technology, revealing great differences for adaptation of bacteria to cold.

The COG functional analysis performed on the genome of the two strains revealed at least three main differences: (i) the first one is related to the COG X category (‘No functional class found’), where PhTAC125 or PspTB41 share about 15% of orthologous genes, accounting for about 45% of the genes specific for the two strains (Fig. 1). (ii) The COG category L (‘Replication, recombination, repair’) was particularly enriched in the PspTB41 genome. Since PspTB41 genome embeds almost 200 MGEs more than PhTAC125 within the L category, it might suggest how this strain is apparently more prone to exchange genetic material than PhTAC125. However, this may be related either to the larger genome size of PspTB41 compared to PhTAC125 and to the fact that the two strains were isolated from different niches. In fact, it was reported that deep-sea bacteria usually have more transposable elements than bacteria in surface sea water^45^. (iii) The genome of PhTAC125 exhibited a significant enrichment of genes belonging to the I category (lipid transport and metabolism), compared to PspTB41. This might be in agreement with the phenomic data showing that PhTAC125 strain is apparently better adapted to cold than PspTB41 (Fig. 2), and with the finding that the increase of genes involved in the synthesis of lipids might result in a better cold-adaptation^20^. Also, the metabolic modeling of the response of PhTAC125 to a temperature downshift has revealed that fatty acids metabolism is particularly active in this specific cellular functional state^32^.

The comparative PM analysis of the growth/no-growth response of PhTAC125 and PspTB41 at 4 °C and 15 °C, provided interesting differences (Fig. 2). The better response of PhTAC125 in terms of metabolic activity onto PM 4–8 plates at 4 °C, might be likely related to protein synthesis metabolism (transcription, translation), which was shown to be remarkably upregulated in PhTAC125 at low temperatures^20, 26, 27^. In particular, comparing the ability of the two strains to utilize specific sources of PM 4–8 plates at 4 °C and 15 °C (Fig. 3), both PhTAC125 and PspTB41 showed a similar (low) metabolic activity in using carbohydrates and other C sources (PM1, PM2 plates), regardless the temperature settings. This result was not surprising as, in contrast to many γ-proteobacteria, *P*. *haloplanktis* do not possess a phosphoenolpyruvate-dependent phosphotransferase system for the transport and first metabolic step of carbohydrate degradation^20^, accounting for their lack of growth on glucose. Nevertheless, each strain revealed a higher metabolic ability to use carbohydrates at 4 °C than at 15 °C.

When grown at 4 °C, PhTAC125 displayed a better response to N-source growth enhancement compared to PspTB41; in particular, it could better utilize many dipeptides and some amino acids such as Glutamate, Glutamine, Citrulline, Asparagine, Serine, Aspartic acid, Threonine, Alanine and Arginine, most of which are known to play a role in cold adaptation mechanisms^4, 46, 47^. Notably, it was also reported that PhTAC125 proteome is enriched in N-residues compared with mesophilic and thermophilic counterparts and, for instance, it is possible to discriminate proteins in PhTAC125 according to their Asparagine (N) content^20^. Furthermore, this aminoacid carries an amide group that is extremely sensitive to temperature and this same Asparagine-driven bias has been reported in other psychrophiles, such as *Psychromonas ingrahamii* ^48^, providing a rationale for an Asparagine excess in psychrophiles. However, the role of Asparagine excess in proteins still deserves further investigations in order to unravel if and how the organism regulates various proteins in response to cold temperatures.

The fact that PhTAC125 can more effectively utilize sulfur and phosphorous sources than PspTB41 at 4 °C, other than N compounds, confirms its enhanced ability to synthesize aminoacids and proteins under cold temperatures. Nevertheless, as literature reports a cellular decrease of the phosphate content in PhTAC125 under cold temperatures^29^, it might likely enhance the uptake of new phosphate from the environment. Furthermore, phosphorous availability is also essential for the bacterial regulatory systems and signaling. In fact, annotation of the PhTAC125 genome highlighted the presence of a large number of regulatory mechanisms including typical two-component systems^20^, which are known to have an important role in the adaptation of bacteria to deep-sea conditions^14^. For example, Histidine kinase response regulator systems - one of the major stimulus perception/signalling transduction cascades in bacteria - are more enriched in deep sea water microbes than in surface sea water microbes^45^. This shows that signal transduction genes have an important role in the adaptation of bacteria to deep-sea conditions. PhTAC125 contains 7 histidine kinase genes whereas PspTB41 just 2 (data not shown). Thus, it is possible to argue that such signal transduction genes might have a key role in the adaptation of bacteria to cold environments as well.

A considerable number of substrates metabolized by PhTAC125 at 4 °C belonging to the “chemicals” KEGG category are biocides or antimicrobial compounds, suggesting a higher resistance of PhTAC125 to such molecules at low temperatures compared to PspTB41 (75 vs 51, respectively). This might be quite surprising, as PspTB41 was isolated from a marine sponge (*Anoxycalyx joubini*), which are known to produce substances that have antimicrobial activity^49^; accordingly, PspTB41 was expected to be more resistant than PhTAC125 to antimicrobials. However, sponges do not live at extremely cold temperatures. Thus, it is possible that sessile sponge-associated organisms such as PspTB41 are not able to activate its resistance mechanisms at cold temperatures (4 °C); in fact, when grown at 15 °C, PspTB41 exhibited higher resistance to antibiotics and other toxic compounds than PhTAC125 (98 vs 26, respectively).

Most of the different metabolic response of PhTAC125 and PspTB41 to cold temperatures is supposed to be related to their capability to cope with oxidative stress. For example, the presence of *ygfZ* (PSHAa0722), *dfrA1* genes (PSHAa2653) and two GST-like genes (*yibF*) only within the genome of PhTAC125 might justify its better response to oxidants under cold temperature compared to PspTB41. This was confirmed experimentally, for instance, on Plumbagin, 3, 4-Dimethoxybenzyl alcohol or Methyl viologen (Table S2).

On the other hand, despite the ability of PhTAC125 and PspTB41 to grow at 15 °C under a wide range of NaCl (1–10%) and KCl concentrations (3–6%), PhTAC125 exhibited strong growth limitations under cold temperature compared to PspTB41. Thus, although the genomic potential of both PhTAC125 and PspTB41 to grow under osmotic stress was verified at 15 °C, it is quite surprising observing how their growth under such osmotic conditions is remarkably sensitive to temperature, suggesting a close relationship between salt and cold adaptation. Remarkably, it should be noted that: (i) the salt concentration affects both the freezing temperature of the water and the stability of enzymes; (ii) glutathione has been found to play, in bacteria, a key role as antioxidant but also as detoxification agent in the response to salt stress^50^. Interestingly, despite most of the genes coding for the enzymes involved in the Glutathione metabolism are shared between PhTAC125 and PspTB41, except for trypanothione synthetase (3.5.1.78) and glutathionyl-spermidine synthase (6.3.1.8), the catabolism of several substrates such as Glutatione (GSH), L-Cysteine, Glycine, Putrescine, L-Ornithine, Spermidine and Spermine is strongly reduced in PspTB41 at 4 °C compared to PhTAC125 (Figure S1). This result indicates that PspTB41 strain is not able to efficiently use such substrates under cold conditions and it might be due to a reduced activity of the enzymes directly involved in the reactions^44^. Interestingly, together to GSH, glutathionylspermidine is likely to be a parallel defense strategy of PhTAC125 against oxidative stress and has been proposed to be more effective at preventing DNA damage induced by free radicals or oxidative species than GSH. Furthermore, S-glutathionylation is considered one of the major redox-regulatory mechanism in eukaryotes and protects active site cysteine residues against overoxidation to sulfonic acids^41^. First studies identified S-glutathionylated proteins also in Gram-negative bacteria and more recently it was found also in *P*. *haloplanktis* where it modulate the *in vivo* function of SOD enzyme at low temperatures, while protecting the active site from damage in an easily reversible manner^51^. However, the regulatory role of protein S-thiolation for bacterial physiology has only recently been investigated^41^. Thus, we speculate that protein S-thiolation might be a key redox regulator in PhTAC125 and a possible mechanism for cold adaptation. Such hypothesis is consistent with its enhanced catabolism of S compounds (PM4) and Ductape results. In fact, at 4 °C the substrate with the highest AV value is Spermine (KEGG code C00750), which is directly involved in two metabolic pathways differentially related to cold-adaptation mechanisms: (i) ‘Glutathione metabolism’ (KEGG map480), which has a key function in protecting the cell from the action of low pH, chlorine compounds and – most of all - oxidative and osmotic stress^41, 52^. The fact that Spermine was better catabolized by PhTAC125 than PspTB41 at 4 °C and the lack of glutathionyl-spermidine synthase genes in PspTB41, implying that PspTB41 typically faces low ROS concentrations, suggested an increased amount of highly active antioxidative enzymes which likely helps PhTAC125 to cope with oxidative stress in the cold environments; (ii) ‘Arginine and Proline metabolism’ (KEGG map330), which was previously reported to be related to bacterial cold-adaptation strategy^9^. In fact, these aminoacids form multiple hydrogen bonds and salt bridges and their presence in proteins is related to a decrease in the conformational flexibility. Accordingly, psychrophilic enzymes show reduced levels of such amino acids. Indeed, PspTB41 lacks of the Ornithine cyclase gene (EC 4.3.1.12), which provides Proline and ammonia via cyclization of an ornithine molecule. Furthermore, despite L-Proline substrate is efficiently used as C-source at 15 °C, it was shown to be better metabolized by PhTAC125 as N-source or nutritional supplement, compared to PspTB41. L-Ornithine is also involved in the Glutathione metabolism and resulted to be one of the substrates indicated to be responsible of the better cold-adaptation of PhTAC125 compared to PspTB41 (Fig. 5a). Furthermore, Arginine catabolism could also provide a direct source of ammonia under nitrogen- limiting conditions, through the AST pathway, while providing metabolites for cold adaptation^53^.

Citrulline is another substrate related to the ‘Arginine and Proline’ metabolism and ‘Biosynthesis of amino acids’ (KEGG map1230), which was better metabolized by PhTAC125 than PspTB41 as both N-source and nutritional supplement. It is the product of the ornithine carbamoyl transferase (EC 2.1.3.3) that catalyzes the conversion of Ornithine and Carbamoyl phosphate into Citrulline and inorganic phosphate, and was shown to display high activity also at low temperatures in psychrophilic bacteria^54^.

Pyridoxine, involved in vitamin B6 metabolism, also displayed a high AV value and was better metabolized by PhTAC125 than PspTB41. Interestingly, this result is consistent with the anabolic functions and amino acid metabolism of Pyridoxine previously observed under cold conditions in other organisms^55^. Furthermore, the ability of PhTAC125 to grow at 4 °C on Caprylic Acid (related to fatty acid biosynthesis) is consistent with the predicted higher fluxes of reactions involved in fatty acid biosynthesis/metabolism following a cold shock, as reported in a recent paper^32^. This result confirms the important role of fatty acid metabolism in bacterial cold adaptation^56^.

Another substrate which highlighted high AV value was Aspartate, the substrate of Aspartate aminotransferase, a previously-characterized psycrophilic enzyme^57^, and which was more efficiently used by PhTAC125 than by PspTB41.

Finally, PhTAC125 better utilized L-Serine, L-Threonine, L-Valine and Glycine than PspTB41 as N-source or nutritional supplement, confirming the enhanced capability of PhTAC125 to cope with cold through the uptake of such amino acids which are known to increase protein conformational flexibility^9, 58, 59^.

On the other hand, relevant metabolic differences were further highlighted between PhTAC125 and PspTB41 when the strains were compared at 15 °C (Fig. 5b). In contrast with the results observed at 4 °C, here the main metabolic differences were differentially expressed by the two strains (Fig. 5b) and appeared be more related to osmotic stress than cold adaptation. For example, Trigonelline and Glycine Betaine (AV = 7) showed the highest AV values in PspTB41 under osmotically stressed conditions (NaCl 6%) which are limiting for PhTAC125; interestingly, trigonelline is involved in the ‘Nicotinate and Nicotinamide’ metabolism (KEGG map760) which provide essential cofactors in enzyme-catalysed reduction-oxidation reactions. As nicotinamide (vitamin B3) is an effective antioxidant against oxidative damage. Glycine Betaine is a highly effective osmolyte in many bacteria and it was reported to be key mediator of the cold-tolerant phenotype in some bacteria, such as *Listeria monocytogenes* ^60^ and *Synechococcus* sp.^61^. Thus, its high AV values in PspTB41 at 15 °C might explain the better adaptation of such strain to osmotic stress as compared to PhTAC125. Furthermore, even though PspTB41 was able to better catabolize Glutathione, PhTAC125 displayed a better growth on Putrescine, Cadaverine and N-Acetyl-D-Glucosamine substrates, which are involved both in ‘Glutathione metabolism’ (KEGG map480) and in ‘ABC transporters’ (KEGG map02010) specific for peptides and lipids. Such compounds are key elements in the biosynthesis of molecules involved in common cold-adaptation strategies of bacterial cells^9^. Most of the other compounds with high AV value are related to high osmotic adaptation (NaCl 6%) or similarly catabolized at 4 °C, even if at a lower rate. Thus, they will be not considered involved in the cold-adaptation strategy but as simply related to specific features which differences PhTAC125 and PspTB41.

**Figure 6:**
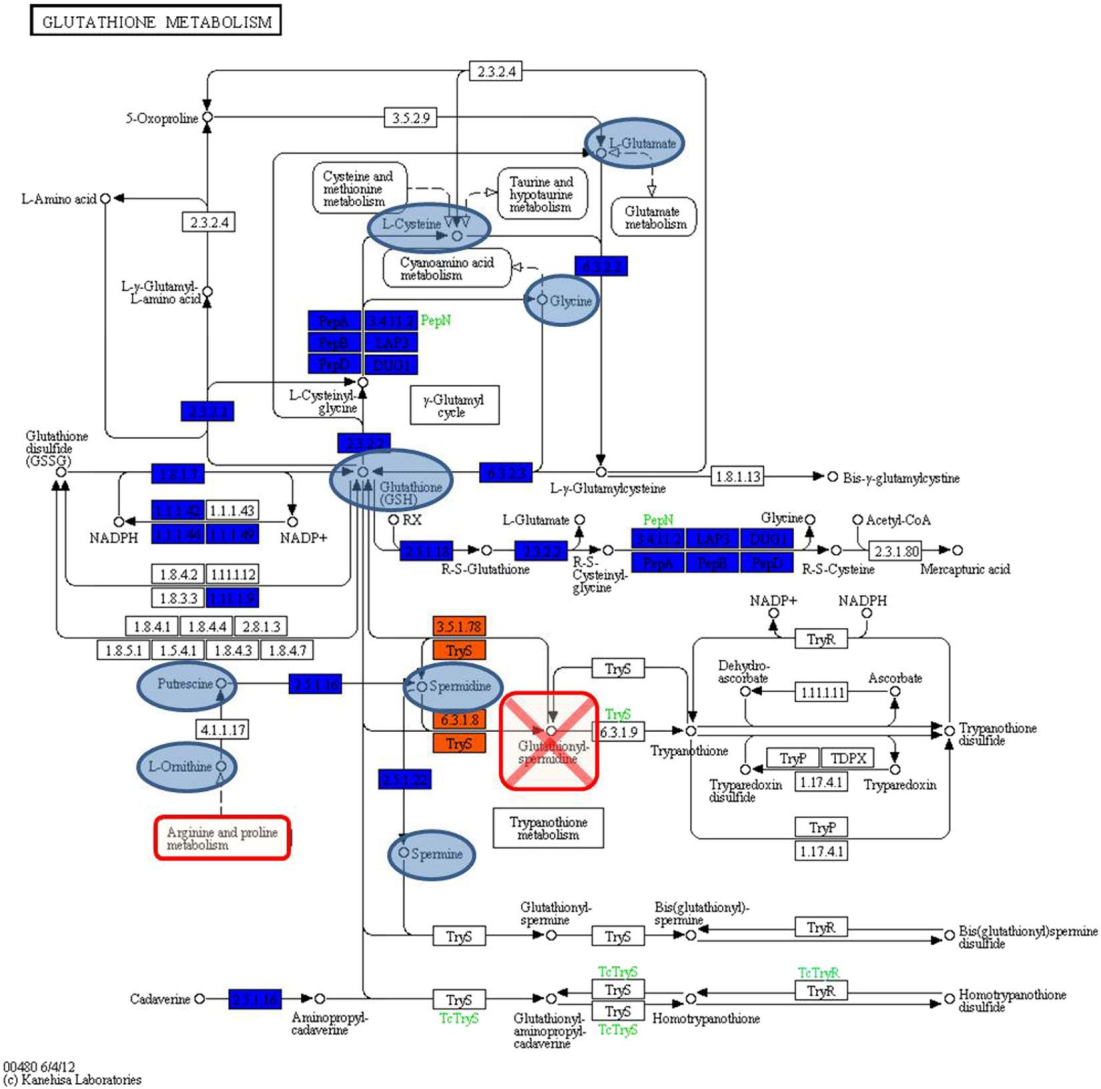
Glutathione (GSH) metabolic network analysis using the dape module (KEGG map00480, reproduced with permission of Kanehisa Laboratories, Japan). Boxes represent reactions while small circles represent compounds. Core reactions are colored blue, variable reactions are colored orange; blue circles represent compounds better used by PhTAC125 at 4 °C, compared to PspTB41. The orange reactions indicates that PspTB41 is not able to carry out reactions catalyzed by glutathionylspermidine amidase/synthetase (3.5.1.78) and glutathionylspermidine synthase (6.3.1.8), resulting in its inability on producing glutathionylspermidine and S-thiolation. Gluathione metabolism is strictly related to ‘Arginine and Proline metabolism’.

Overall, the ‘multi-omic’ approach here reported provided an accurate evaluation of the metabolic pathways putatively involved in the cold adaptation mechanisms of PhTAC125 and PspTB41 strains; despite its significantly larger genome size, PspTB41 displayed a reduced metabolic adaptation to cold temperatures (4 °C) compared to PhTAC125. In contrast, PspTB41 appeared to be much more competitive when grown at 15 °C, revealing a remarkable adaptation to high osmotic stress. A list of the possible genomic/metabolic features involved in such process provided evidence of the capacity of PhTAC125 to enhance the uptake (or the catabolism) of compounds at 4 °C that may confer cryotolerance, including Spermine, Glutathione, Ornithine and other compounds related to ‘Glutathione metabolism’ (Fig. 6). Moreover, ‘Arginine and Proline metabolism’, ‘β-Alanine metabolism’ and ‘Fatty acid biosynthesis’, might also explain its better cold adaptation than PspTB41. Indeed, the increased metabolic potential in terms of fatty acid uptake and biosynthesis in PhTAC125 confirmed to be just one of its adaptive strategies to cold environments whereas protein S-thiolation appears to be one of the main strategies for cold adaptation in PhTAC125. Thus, we have provided an example of how multi-omics information can be used for connecting microbial genotype(s) to their underlying phenotype(s) and a promising approach for future experimental characterization of cold-related processes in bacteria.

## Methods

### Bacterial strains and genome sequences

PhTAC125 was isolated in 1992 from seawater near the French Antarctic Station Dumont d’Urville (60°40′; 40°01′E)^62^. PspTB41 was isolated from the marine sponge *Anoxycalyx joubini* at Terra Nova Bay (Ross Sea) during the XX Italian Expedition to Antarctica (Austral Summer 2004–2005)^63^. Both strains were grown on TYP medium and maintained at −80 °C in cryovials with 40% of glycerol. PspTB41 belongs to the Italian Collection of Antarctic Bacteria (CIBAN) of the National Antarctic Museum (MNA).

The genome sequences of PhTAC125 and PspTB41 were obtained from previously published works^20, 32^ and were here post-processed and analyzed using the Ductape suite^64^. They are available in GenBank with accession codes NC_007481.1 and NC_007482.1 for PhTAC125 and AUTH00000000.1 for PspTB41. Briefly, the main genomic features of the two strains: the PhTAC125 strain has 2 replicons, a genome size of 3850272 bp, 3484 ORFs and GC% = 40.09. The PspTB41 strain has 122 contigs, a genome size of 4632606 bp, 4217 ORFs and GC% = 40.34.

### Phenotype microarray analysis

The BIOLOG Phenotype Microarray (PM) technology, comprising a total of 1920 unique tests, uses tetrazolium violet irreversible reduction to formazan as a reporter of active metabolism^65, 66^. PhTAC125 and PspTB41 strains were tested on twenty 96-well PM microplates (PM 1–20) comprising different metabolic and toxic compound conditions, including 192 assays of C-source metabolism (PM 1–2), 384 assays of N-source metabolism (PM 3, 6–8), 96 assays of P-source and S-source metabolism (PM 4), 96 assays of biosynthetic pathways (PM 5), 96 assays of ion effects and osmolarity (PM 9), 96 assays of pH effects (PM 10), and sensitivity to 240 chemicals (PM 11–20) (see Supplementary information and Table S1 for the detailed procedure).

Usually, all the PM plates are simultaneously incubated into the Omnilog Reader (Biolog) under controlled temperature over few days, depending on the strain. However, as the native Omnilog instrument has an operating temperature range between room temperature and 45 °C, two sets of PM microplates for each strain were prepared and separately incubated at 4 and 15 °C into alternative standard temperature incubators. Thus, the metabolic activity was manually assessed on a Synergy HT microplate reader (BioTek Instruments Inc., USA), three times a day, until the color development reached the plateau at 4 and 15 °C after 194 h and 167 h, respectively. The cellular growth was determined by measuring the absorbance lengthwave (OD 590 nm) values of the kinetic curve. The output data were analyzed using the DuctApe suite as described in the next section. Negative control wells, containing the inoculated growth medium but lacking the C-source, were also measured to normalize differences in inocula and redox dye oxidation between samples. In particular, OD = 0.45 was selected as cut-off value for cellular growth within each well of the PM microplates as tetrazolium color development was visually detected only for OD >0.45.

### Linking genomes to phenomes: the ductape analysis

Available genomic and experimental datasets of the two strains were post-processed and analyzed with the DuctApe suite (v. 0.16.2)^64^, using the default settings. Briefly, the three analyses performed with the DuctApe software were:

- Comparative analysis of the genomes of strains PhTAC125 and PspTB41 (dgenome module);
- Comparative analysis of the phenomic data (BIOLOG PM data) of strains PhTAC125 and PspTB41 (dphenome module);
- Metabolic reconstruction according to the KEGG database^67^ (dape module), using the outputs of the previous two analyses as input;

The dphenome module of the DuctApe software calculates a parameter for each well (activity index, AV), that provides both qualitative (presence/absence) and quantitative information on the ability to metabolize a compound. Briefly, AV values range from 0 (red) to 9 (green) and indicate those wells exhibiting low or no metabolic activity (AV = 0) and those with high metabolic activity (AV = 9), respectively. The AV parameter is calculated through a k-means clustering (with k clusters) on five growth curve parameters (max, area, average height, lag time, and slope). More details on the calculation of the AV can be found in Galardini *et al*.^64^. Wells showing AV values that differed more than 3 units have been used as outputs for the metabolic reconstruction through the dape module. In particular, the dphenome module output may be also visually reported as ‘Activity rings’, where each strip is colored according to the calculated Activity Index (AV) and accounting for the observed growth kinetics of each strain on that specific source. From this output is possible to manually screen up all the substrates, one by one, in order to highlight any putative correlation with any cold-adaptation-related mechanism.

Finally, the “dape” module is used to combine the data gathered by the other two modules and provide insights into the genetic determinants of the observed phenotypic variability, using the KEGG database as the metabolic information source. The pathways and PM compounds are ranked by their genetic and phenotypic variability, respectively.

## Electronic supplementary material

Supplementary material

Table S2
