# Supplementary material for: Ecology of cold environments: new insights of bacterial metabolic adaptation through an integrated genomic-phenomic approach
Source: Sci Rep. 2017 Apr 12;7:839. doi: 10.1038/s41598-017-00876-4 (PMC5429795; doi:10.1038/s41598-017-00876-4)
Supplement: Supplementary file 1 — Supplementary material [file 41598_2017_876_MOESM1_ESM.doc]

**Supplementary information**

**Ecology of cold environments: new insights of bacterial metabolic adaptation through an integrated genomic-phenomic approach**

Stefano Mocali, Carolina Chiellini, Arturo Fabiani, Silvia Decuzzi, Donatella de Pascale, Ermenegilda Parrilli, Maria Luisa Tutino, Elena Perrin, Emanuele Bosi, Marco Fondi, Angelina Lo Giudice, Renato Fani

**Document S1 - Phenotype microarray analysis: detailed procedure**

*Bacterial growth conditions*

In order to select the optimal growth medium for the phenotype microarray (PM) analysis, PhTAC125 and PspTB41 strains were preliminary plated on both Marine Agar 2216 medium (MA, Difco) and TYP agar medium (16 g/l yeast extract, 16 g/l Bacto-tryptone, and 15g/l NaCl at pH 7,5). Cells were then swabbed from the plates after overnight growth and suspended in appropriate medium containing Schatz salts (NaCl 10g/l, KH_2_PO_4_ 1g/l,NH_4_NO_3_ 1g/l, MgSO_4_ 7H_2_O 0.2g/l, FeSO_4_ 7H_2_O 10mg/l, CaCl_2_ 2H_2_O 10mg/l) as additive solution until the 85% transmittance suspension of cells was obtained on a Biolog turbidimeter. The obtained suspension was inoculated in 96-well microplates Ecoplates^TM^ (BIOLOG), which contain 31 different substrates as C-source, and incubated at 4°C for about 8 days. The colorimetric assay was considered as positive when the absorbance value (Optical Density at 590nm) corresponding to the reduced dye (indicating substrate utilization) was at least 0.365. This threshold was chosen based on the calculation of standard deviations of the absorbance of the reduced dye in the negative control wells (S.D. was about 8%) and which corresponded to the color appearance detectable by eye.

The same procedure was adopted for the PM assay. In this case 0.4% succinate was used as C source for the inoculation of PM plates (when appropriate) at 4°C and 15°C for about 8 and 7 days, respectively. More details of the procedure are reported in the next section.

*Preliminary assays and growth conditions*

A preliminary comparison between PhTAC125 and PspTB41 growth curves was carried out on TYP medium under low (4°C) and permissive (15°C) temperatures. The two strains showed a different ability to grow at both temperatures. In particular, at 15°C both PhTAC125 and PspTB41 provided a significant increase of growth rate (OD_600_) compared to 4°C, according to previous studies. Furthermore, within the first 12h of incubation, PhTAC125 expressed a higher growth rate compared to PspTB41 at both temperatures, confirming to be one of the most versatile and fastest growing psychrophiles characterized so far. This result confirmed also that, despite taxonomically closely related, PhTAC125 and PspTB41 display a different growth response at 4°C and 15°C, thus representing an optimal case study to compare the different intraspecific molecular strategies to cold adaptation.

A fundamental prerequisite to properly compare the phenome of two different strains through the Omnilog^TM^ Phenotype MicroArrays is making their growth condition as similar as possible under the same experimental conditions. In order to do that, PhTAC125 and PspTB41 strains were grown overnight at 20°C in both MA and TYP agar medium. Then, they were inoculated unto BIOLOG Ecoplates^TM^ (similar to PM plates but containing 31 different C-sources) and incubated at 4°C in order to compare their growth rate at low temperatures in such experimental conditions. Values of absorbance (OD_590_) recorded for the two strains grown in each culture media are reported in table S1. When the OD≥0.365 (the minimum OD value related to a visible color development which was chosen as metabolic “threshold”) the wells developing color are highlighted in pink with the time required to get colored (r) expressed in hours. The wells with absorbance values not shared between the two strains are highlighted in purple, whereas the well highlighted in blue indicates the substrate which only PhTAC125 could metabolize at 4°C (table S1). Although the two strains showed a different metabolic profile, their growth rate was comparable and the catabolic activity on such C-sources appeared quite similar regardless they were grown in MA or TYP medium. The two strains showed a similar but not identical catabolic profile: they shared the metabolic responses of 26 substrates out of 31, whereas 4 compounds were preferentially used by TAC125 (L-Phenylalanine, D-Mannitol, alfa-Cyclodextrine and L-Threonine) and 1 by TB41 (Acetyl-Glucosamine). These results suggested a higher versatility of PhTAC125 metabolism at 4°C and its adaptive potential to better use C-sources compared to TB41 under cold conditions. Interestingly, PhTAC125 grown on TYP medium was able to better catabolize L-Serine (Table 1, in blue) than PhTAC125 grown on MA. Furthermore, despite the shared substrates were generally metabolized faster by PhTAC125 than PspTB41 (es. Pyruvic Acid Ester, Tween 40 and Tween 80), the color development rate of the two strains at 4°C was comparable. Thus, we decided to continue the analysis by using TYP as growth medium.

Despite strain PhTAC125 is well-adapted to fast growth, it does not possess a phosphoenolpyruvate-dependent phosphotransferase system for the transport and first metabolic step of carbohydrate degradation (Medigue et al., 2005). This accounts for its lack of growth on glucose and other C-sources, as confirmed also by the results of the preliminary Ecoplate assay. Therefore, different easily metabolized C-sources (glutamate, succinate, malate, pyruvate) were supplied to PhTAC125 and PspTB41 suspensions (inoculation fluid) as unique C source and compared at 4°C in order to select the best C source for both; the results suggested that 0.4% succinate was the best choice (data not shown).

*PM microplate inoculation*

The cells were then picked up with a sterile cotton swab and suspended in 15 ml inoculation fluid (IF-0, Biolog) with Schatz salts (NaCl 10 g l^−1^, KH_2_PO_4_ 1g l^−1^, NH_4_NO_3_ 1g l^−1^, MgSO_4_ x 7H_2_O 0.2 g l^−1^, FeSO_4_ x 7H_2_O 10 mg l^−1^, CaCl_2_ x 2H_2_O 10 mg l^−1^) as additive solution and 1% (v/v) tetrazolium violet (Dye Mix A, Biolog). Cell density was adjusted to 81% transmittance (T) on a Biolog turbidimeter. Then, 100-μL aliquots were added to carbon-source plates (PM1 and PM2A). For all the other microplates (PM3-20), inoculations were supplemented with 0.4% succinate as C-source.

**Table S1.** Metabolic activity on different C sources in EcoPlate^TM^ microplates espressed as adsorbance (OD_590_ nm) of PhTAC125 and PspTB41 strains after a pre-inoculation at 4°C on Marine agar (MA) and TYP agar (TA) over-night. The number of hours required to develop a visible color are also reported (r). The substrates that provided a color development comparable between the two strains were highlighted in light pink; the substrates that provided a color development differentially expressed between the two strains were highlighted in purple; the substrates that provided a color development specific for one of the two strains were highlighted in blue.

**Table S2:** Full Biolog data of PM1-20 of the TAC125 and TB41 strains under 4° and 15°C, reported as adsorbance (OD=590nm)

See separate Excel document

**Table S3 –** PM substrates used by PhTAC125 at 4°C but not at 15°C

**Table S4 –** PM substrates used by PspTB41 at 4°C but not at 15°C
